# Pediatric liver cirrhosis interventional procedures: from biopsy to transjugular intrahepatic portosystemic shunt

**DOI:** 10.1007/s00247-022-05492-7

**Published:** 2022-09-19

**Authors:** Gian Luigi Natali, Giulia Cassanelli, Guglielmo Paolantonio, George Koshy Parapatt, Lorenzo Maria Gregori, Massimo Rollo

**Affiliations:** 1 Interventional Radiology Unit in Oncohematology, Department of Imaging, Bambino Gesù Children’s Hospital, IRCCS, Piazza S. Onofrio, 4, 00165 Rome, Italy; 2 Interventional Radiology Unit, Bambino Gesù Children’s Hospital, IRCCS, Rome, Italy; 3 Imaging Department, Bambino Gesù Children’s Hospital, IRCCS, Rome, Italy

**Keywords:** Biopsy, Children, Cirrhosis, Embolization, Interventional radiology, Liver, Portal hypertension, Transjugular intrahepatic portosystemic shunt

## Abstract

Cirrhosis is a complex diffuse process whereby the architecture of the liver is replaced by abnormal nodules because of the presence of fibrosis. Several pediatric diseases such as extrahepatic portal vein obstruction, biliary atresia, alpha-1-antitrypsin deficit and autoimmune hepatitis can lead to cirrhosis and portal hypertension in children. In this article the authors describe interventional radiology procedures that can facilitate the diagnosis and treatment of diseases associated with liver cirrhosis and portal hypertension in the pediatric population. These procedures include image-guided liver biopsy, mesenteric–intrahepatic left portal vein shunts, balloon-occluded retrograde transvenous obliteration, transjugular intrahepatic portosystemic shunts and splenic embolization.

## Liver biopsy

Liver biopsy is a fundamental tool in the wide field of pediatric liver cirrhosis and can be considered the gold standard procedure to obtain a liver sample for histopathological examination, supporting diagnosis, management and prognosis of many pediatric acute and chronic liver diseases. Liver biopsy can be performed in either native or transplanted liver for diagnostic and prognostic purposes, for assessing the severity of known disease and for monitoring disease progression or response to therapy [1]. Liver biopsy can be performed percutaneously with US guidance, or via a transjugular approach, depending on the clinical status and laboratory results of the pediatric patient.

The first step in liver biopsy is to perform an abdominopelvic US examination to look for the presence of free intraperitoneal fluid (above all in perihepatic recesses), colonic interposition or marked dilatation of the biliary tree and to determine the best site of access to liver parenchyma, which may be subcostal or intercostal. The next step is to determine the international normalized ratio (INR) (normal range 0.9–1.2) and platelet count (normal > 60,000 U/mm^3^) and ensure that the child is not on anti-aggregant or anticoagulant therapy at the time of the procedure. An abnormal coagulation status is an absolute contraindication to performing percutaneous liver biopsy.

## Percutaneous liver biopsy

Percutaneous liver biopsy is performed under US guidance for real-time visualization and orientation of the biopsy needle, using a 16- to 18-gauge (G) needle (BioPince; Argon Medical Devices, Plano, TX) with a 13-mm to 23-mm range and usually one needle pass; more needle passes may be performed when examining a single nodular lesion.

Abdominopelvic US evaluation is done immediately after the biopsy to detect any potential complication, because perihepatic fluid collections and any intra-abdominal free fluid that was absent in the pre-procedural US examination are considered bleeding complications [2].

The child is monitored for at least 6 h after the procedure, with blood evaluation repeated 2 h after the end of the biopsy to look for any hemodynamic changes and, in cases of pain or other symptoms, to carry out a control US examination.

The European Society for Paediatric Gastroenterology Hepatology and Nutrition (ESPGHAN) defines minor complications of liver biopsy as the presence of pain, subcapsular bleeding that does not require transfusion or prolonged hospitalization, infection, minor bile leak or hemobilia and arteriovenous fistula [1]. ESPGHAN defines major complications as bleeding or hemobilia requiring transfusion, need for surgery or intensive care management, pneumothorax, hemothorax or death. Minor complications are immediate and bleeding-related and occur in a reported 4.6–25% of patients [3–7]. These normally do not require intervention, except when there is prolonged local compression or a longer monitoring and a control US evaluation. These kinds of complications are usually related to the number of needle passes during the procedure, age younger than 3 years, body weight <16 kg and lower INR or platelet count [8]. Additionally, transient pain has been noted in 20–36% of pediatric patients after liver biopsy [8, 9]. Liver biopsies performed on transplanted liver are associated with a lower bleeding risk [2]. Several reports have described an incidence of major complications ranging from 0% to 4.6% [3, 5–7, 9, 10], so pediatric percutaneous liver biopsy is considered a safe procedure with a high diagnostic yield [2]. However, in children with coagulation anomalies or ascites, percutaneous liver biopsy is associated with a high risk of hemoperitoneum, sometimes life-threatening. To avoid major complications, a transjugular biopsy performed by an experienced interventional radiologist team is a satisfactory and better tolerated option, although it entails major costs and longer periprocedural time [11].

## Transjugular liver biopsy

Performing a biopsy via the venous system reduces the risk of bleeding because the Glisson capsule is not perforated [12, 13] and, if bleeding does occur, it returns promptly into the venous system rather than into the peritoneum. In general, indications for transjugular liver biopsy are the contraindications to percutaneous biopsy: high prothrombin level, platelet count less than < 60,000 U/mm^3^, INR > 1.5, presence of abundant intraperitoneal fluid and anti-aggregant or anticoagulant therapy that cannot be discontinued. Some authors consider other indications for this procedure, even if coagulation abnormalities or ascites are not detected, including previous unsuccessful percutaneous biopsy, morbid obesity, atrophic liver, suspected amyloidosis, cardiac liver, hemodialysis and chronic renal insufficiency, peliosis hepatis and hereditary hemorrhagic telangiectasia, which all increase bleeding risk [14, 15].

A contraindication to transjugular liver biopsy is the presence of thrombosis in the right internal jugular vein. Contralateral or external jugular, or femoral vein approaches are riskier than the right internal vein approach, and therefore are used as a last resort. Other contraindications to this biopsy approach are thrombosis of hepatic veins, hydatid cysts and cholangitis [14].

The first step in the transjugular liver biopsy procedure is to perform an 18-G (Cook Medical, Bloomington, IN) US-guided right internal jugular vein puncture using a superficial high-frequency linear probe, introducing a 0.035-in. guidewire (Terumo Europe N.V., Leuven, Belgium) and then to insert a 4- to 6-French (Fr) introducer sheath (Terumo Europe) according to the Seldinger technique. The right hepatic vein is then catheterized with a 4- to 7-Fr end-hole catheter (Terumo Europe) and, if necessary, a J-tipped 0.035-in. flexible hydrophilic guidewire (Terumo Europe). An angiography is performed to check that the catheter is in the right position.

**Fig. 1:**
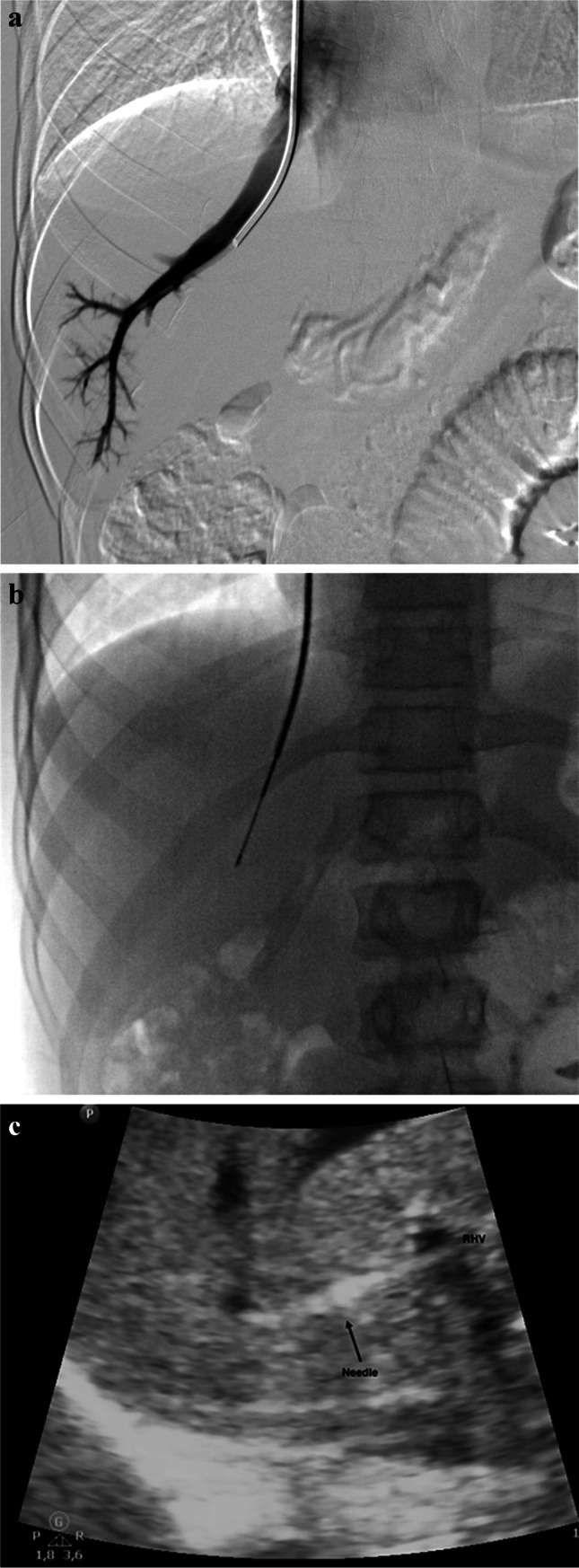
Transjugular liver biopsy in a 9-year-old girl. **a **Angiography posteroanterior (PA) projection shows the correct position of the catheter in the right internal jugular vein. **b** Fluoroscopy PA projection shows the biopsy needle set (14–18 G/7 Fr; Cook Medical, Bloomington, IN) with Colapinto needle open to perform transjugular biopsy. **c** axial US image demonstrates needle guidance during biopsy from right internal jugular vein

Once the interventionist is assured of correct positioning, biopsy is performed under both US and fluoroscopic guidance, using a biopsy needle set (14–18 G/7 Fr) with Colapinto needle (Cook Medical). Cholongitas et al. [16] found that using a semiautomatic system needle core enabled them to obtain a larger, less fragmented and more reproducible liver sample (Fig. 1).

## Portal hypertension

Interventional radiology management of portal hypertension in children usually differs from that in adults [17]. Portal hypertension is defined as an absolute portal venous pressure exceeding 10 mmHg or a pressure gradient between portal and systemic veins greater than 5 mmHg and represents a complication of chronic liver disease or liver vascular occlusion [17–19]. Portal hypertension can have both intrahepatic (pre-sinusoidal, sinusoidal or post-sinusoidal) and extrahepatic (pre-hepatic or post-hepatic) etiologies [17, 18].

In pediatric patients, pre-hepatic portal vein thrombosis is the most common cause of both portal hypertension (∼70% of cases) and upper gastrointestinal bleeding and is usually the result of iatrogenic injury by neonatal catheterization of the umbilical vein or infection (omphalitis), intra-abdominal abscess, sepsis, severe dehydration, abdominal trauma or unknown causes in up to 50% of cases (idiopathic extrahepatic portal hypertension) [17, 20, 21]. Pre-hepatic portal vein thrombosis can result in cavernous transformation of the extrahepatic portal vein with consequent deterioration of portal hypertension and potential development of liver dysfunction, biliary disease, coagulopathy, splenomegaly and ascites [17]. The main target of treatment in pediatric portal hypertension is to prevent the development and bleeding of upper gastrointestinal varices by means of medical therapy, surgical ligation or sclerotherapy of the swollen veins. However, if medical or endoscopic procedures fail, surgical treatment is unavoidable.

Surgical management of portal hypertension has not been well standardized by pediatric liver centers worldwide [19]. While liver transplantation is the major therapy for pediatric patients with primary liver disease resulting in cirrhosis and end-stage liver disease, transjugular intrahepatic portosystemic shunt (TIPS) is frequently considered as a bridge to liver transplantation. Conventional surgical shunts or mesenteric vein–intrahepatic left portal vein shunts (meso-Rex bypass) are preferred for children with pre-hepatic portal vein thrombosis, depending on the vascular anatomy and residual patency [17, 21].

Surgical non-selective portosystemic shunts (such as mesocaval and portocaval shunts) help to reduce portal hypertension but are associated with higher rates of clinical complications such as hepatopulmonary syndrome, encephalopathy or hyperammonemia, which are undesirable in children [17]. Otherwise, the most common selective shunt is the distal splenorenal Warren shunt. The distal splenorenal shunt allows decompression of gastroesophageal varices in both short gastric veins and splenic veins and preserves antegrade perfusion to the liver with less likelihood of clinical consequences [18]. Currently, meso-Rex bypass is the gold standard treatment for pre-hepatic portal vein thrombosis in children with preserved anatomy [21]. Meso-Rex bypass is a venous conduit, usually an autologous graft from the internal jugular vein, connecting the infra-pancreatic superior mesenteric vein to the intrahepatic left portal vein at the Rex recess, the remnant of embryonic umbilical vein. This bypass restores physiological hepatopetal portal flow, avoiding dangerous complications of portosystemic shunting [17, 20, 21].

**Fig. 2:**
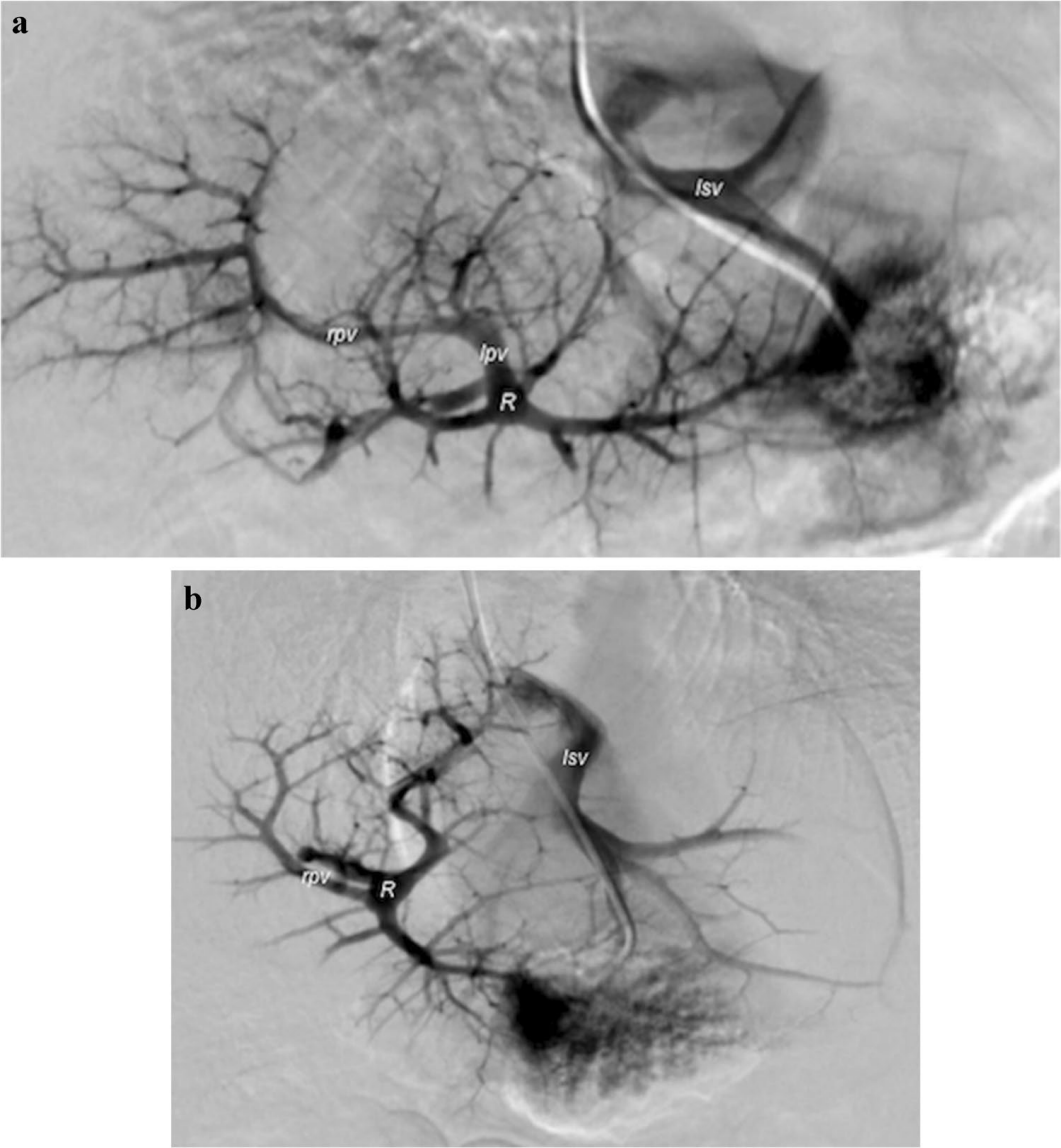
Wedged hepatic venous portography in a 2-year-old girl. **a, b** Digital subtraction images on posteroanterior (PA) (**a**) and right PA oblique (**b**) projections through catheterization of the left suprahepatic vein (*lsv*) show the Rex recess (*R*) and the communication between the left (*lpv*) and right (*rpv*) portal veins

Preoperative imaging is pivotal in the setting of surgical planning. While CT and magnetic resonance (MR) angiography are essential to confirm the pre-hepatic portal vein thrombosis diagnosis and evaluate both the extension of portal cavernoma and size of the extra- and intrahepatic portal system, particularly of the superior mesenteric vein, wedged hepatic venous portography is the mainstay imaging examination for assessing the surgical feasibility of the meso-Rex bypass [17, 20]. Wedged hepatic venous portography consists of retrograde (indirect) phlebography of the intrahepatic portal venous system performed through wedged catheterization of the suprahepatic veins via the right internal jugular vein under general anesthesia. The goal of the procedure is to evaluate the patency of both the Rex recess and left portal vein and to assess the reciprocal communication between right and left intrahepatic portal veins (Fig. 2).

Specifically, Bertocchini et al. [22] categorized the radiologic findings of wedged hepatic venous portography into five specific imaging patterns (A to E), proposing meso-Rex bypass surgery only for children with patent Rex recess (subtypes A to C) and opting for conservative follow-up for clinically stable children or portosystemic shunt creation in cases of complicated portal hypertension. Therefore, this preoperative examination allows the identification of children who are eligible for meso-Rex bypass and thus avoids unnecessary surgical exploration for those with thrombosed Rex recess [22]. Moreover, the hepatic venous pressure gradient can be measured simultaneously to provide important supportive diagnostic information, for instance during the transjugular liver biopsy procedure. Although available data on hepatic venous pressure gradient measurement in the pediatric population are very limited, pressure threshold ≥10 mmHg is predictive of the formation of varices and ≥12 mmHg is associated with decompensation with ascites or variceal bleeding, similar to findings in adults [17, 23, 24]. Although technically feasible, direct portography through transhepatic percutaneous access to the left portal venous system is not recommended because of the risk of vascular complications at the surgical anastomosis site [17].

**Fig. 3:**
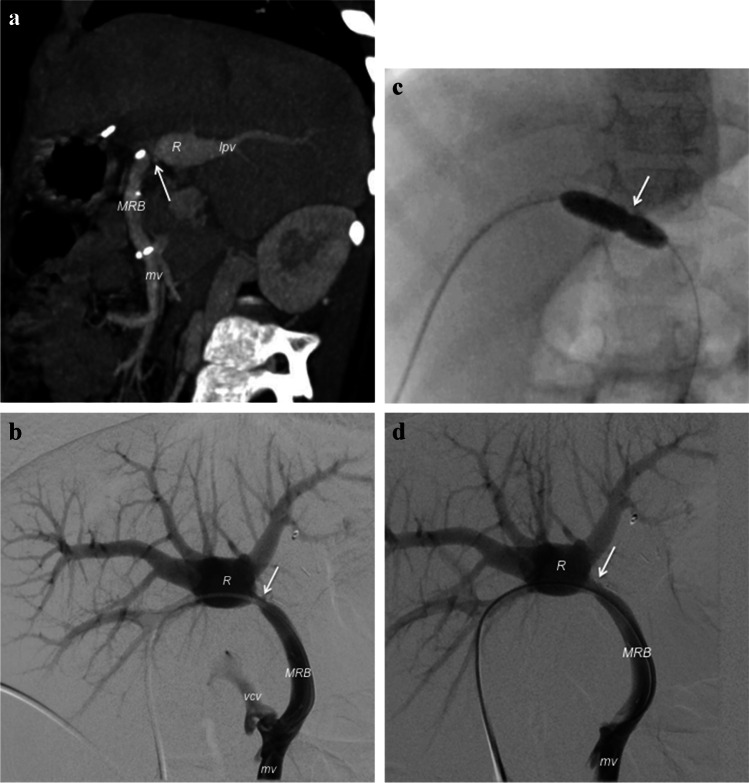
Endovascular treatment of stenotic meso-Rex bypass (MRB) in a 5-year-old girl. **a** Contrast-enhanced multi-detector-row sagittal CT image shows a stricture within the MRB (*arrow*). *lpv* left portal vein, *mv* mesenteric vein, *R* Rex recess. **b–d** Posteroanterior images during percutaneous transhepatic balloon angioplasty (digital subtraction scans in **b** and **d**). Mesenteric portogram (**b**) shows stenosis (*arrow*) within the left portal vein anastomosis of the MRB and vicariant collateral veins (*vcv*) arising from the mesenteric vein before the shunt. Balloon angioplasty of the anastomotic stricture (*arrow*) under fluoroscopic view (**c**). Fluoroscopy (**d**) shows the disappearance of the stenosis and collateral circulation after endovascular dilatation

Meso-Rex bypass is the recommended option for children with pre-hepatic portal vein thrombosis but requires normal liver architecture to ensure long-term patency [24]. Moreover, interventional radiology is usually the first treatment choice in cases of stenosis or occlusion of the shunt through angioplasty, stenting or thrombectomy [17, 21] (Fig. 3). Some authors have also described recanalization of the portal system using different techniques [25–27].

## Balloon-occluded retrograde transvenous obliteration

Bleeding from ruptured esophagogastric varices is one of the most serious complications in children with liver cirrhosis and is a major cause of death in these children [28]. Balloon-occluded retrograde transvenous obliteration is commonly used for the prevention and treatment of bleeding esophagogastric varices in Japan and has also become popular elsewhere in Asia. However, it has only recently gained wider attention in the United States and Europe and is still underused. The procedure is a minimally invasive technique in the treatment of esophagogastric varices, with a reduced risk of rupture or bleeding.

The two main clinical indications for this procedure are active or impending esophagogastric variceal bleeding and esophagogastric varices with hepatic encephalopathy refractory to medical management. On the other hand, the contraindications to balloon-occluded retrograde transvenous obliteration are considerable and include severe uncontrollable coagulopathy associated with liver failure, splenic vein thrombosis, portal vein thrombosis and uncontrolled bleeding from esophagogastric varices. While uncontrolled esophageal variceal bleeding can be considered a contraindication to balloon-occluded retrograde transvenous obliteration therapy alone, TIPS combined with balloon-occluded retrograde transvenous obliteration or balloon-occluded *antegrade* transvenous obliteration is recommended instead. Therefore balloon-occluded retrograde transvenous obliteration can be useful in multiple ways and in combination with other techniques [29].

In conducting balloon-occluded retrograde transvenous obliteration, preprocedural CT imaging is important to document the presence of a portosystemic shunt and assess eventual contraindications such as venous thrombosis. The balloon-occluded retrograde transvenous obliteration procedure in the pediatric population is performed under general anesthesia or deep sedation by interventional radiologists. It consists of endovascular closure of the portosystemic shunt outflow, using an occlusion balloon (Selecon Balloon Catheter; Clinical Supply, Gifu, Japan) followed by injection of a sclerosing agent directly into the gastro-variceal complex. The role of balloon occlusion can be diagnostic or therapeutic. It is useful to perform retrograde venography to visualize the gastric–variceal complex, to modulate flow and to cause stagnation of the sclerosing agent within the gastric–variceal system without reflux of the sclerosant into either the portal or systemic vasculature once occlusion of the shunt has been accomplished. Flow stagnation is helpful to maximize the effect of the sclerosing agent on the gastrovariceal system endothelial lining, leading to thrombosis and subsequent scarring of the system [30].

The use of a sclerosing agent can be selective or superselective and the agents frequently used are ethanolamine-oleate-iopamidol, sodium-tetradecyl-sulfate, polidocanol as foam and N-butyl-2-cyanoacrylate. Microcatheters and embolization coils are adjunctive tools used to administer the sclerosant in high concentration within the varix, minimizing loss of sclerosant into nontarget vascular beds [31].

Possible adverse effects of balloon-occluded retrograde transvenous obliteration include transient ascites, pleural effusion and worsening of esophageal varices. These adverse effects can be caused by elevation of portal pressure in response to occlusion of the portosystemic shunt [29]. Bleeding control rate of gastric varices after balloon-occluded retrograde transvenous obliteration is described as greater than 90%, and therefore this procedure can be attempted in children with a poor hepatic functional reserve and even in children with encephalopathy [32].

Recent reviews of balloon-occluded retrograde transvenous obliteration reported high rates (> 90%) of complete eradication of gastric varices and low rates (< 10%) of gastric variceal recurrence during long-term follow-up compared to endoscopic variceal ligation [33]. Balloon-occluded retrograde transvenous obliteration seems to be an underused treatment modality in children, and more experience in the pediatric field is necessary before recommending its routine use [34].

## Splenic embolization

Splenomegaly is a common condition resulting from cirrhosis. Portal hypertension causes splenomegaly and formation of esophageal varices and a collateral venous circulation [35, 36]. In the clinical context of cirrhosis, splenomegaly is often associated with hypersplenism, a well-known clinical hematologic syndrome caused by an enlarged and overactive spleen and characterized by thrombocytopenia, leucopenia, neutropenia and anemia [37, 38]. In the setting of cirrhosis, thrombocytopenia caused by hypersplenism occurs at frequencies of 64–84% and leukopenia at 5% [37].

Hypersplenism may worsen the course of the disease in children with cirrhosis, because of the increased risk of infection and bleeding, and it may also adversely affect the administration of drugs that could induce leukocytopenia or thrombocytopenia [36, 39]. As mentioned, portal hypertension determines formation of esophageal varices, which, in combination with decreased hematological indices puts children with chronic liver cirrhosis at risk of potential life-threatening bleeding [35, 40].

Surgical splenectomy has been traditionally performed in hypersplenism accompanying chronic liver disease [37, 38]. But although splenectomy is effective in improving hematological indices [37], this surgical procedure in children with cirrhosis carries significant perioperative and postoperative risk [36, 38, 41]. Morbidity from complications after laparoscopic and open splenectomy ranges from 9.6% to 26.6% [40]. Major complications include portal vein and mesenteric vein thrombosis and higher rates of overwhelming sepsis from encapsulated bacteria [37, 39, 40]. As widely documented in the literature, children are particularly vulnerable to post-splenectomy sepsis [42, 43]. For these reasons, operative splenectomy in cirrhotic children with hypersplenism has gradually been discontinued because of the high mortality rate [38].

However, in the last few decades partial splenic embolization has emerged as an excellent alternative to these treatments in the setting of portal hypertension [37, 38, 44]. In 1973, Maddison [45] was the first to describe partial splenic embolization for the treatment of thrombocytopenia and variceal bleeding in cirrhosis [46, 47]. Subsequently, major complications like splenic abscess, splenic rupture, pneumonia and septicemia following splenic embolization were described [36, 47]. In 1979, Spigos et al. [48] described a modified partial splenic embolization approach with limited volume embolization in conjunction with antibiotic prophylaxis and effective postembolization pain control [35]. Spigos et al.’s procedure has been proved to be safe and effective for vascular occlusion [35, 36, 47].

**Fig. 4:**
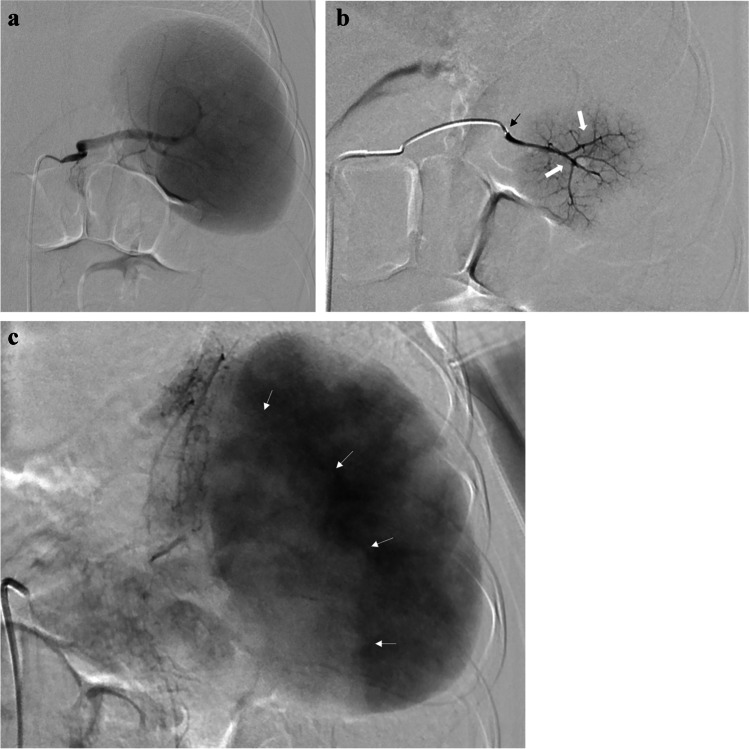
Partial splenic embolization in a 7-year-old girl with portal hypertension and hypersplenism. **a** Splenic artery posteroanterior (PA) pre-embolization arteriogram shows an enlarged and globus spleen. **b** PA selective arteriograms performed before embolization show the microcatheter tip (*black arrow*) and the lower pole arterial branches (*white arrows*) targeted for selective embolization with particles. **c** Post-embolization splenic artery angiogram (PA projection) demonstrates an area of reduced angiographic blush of the lower splenic pole (*arrows*) and the preserved perfusion of the remaining parenchyma

Since then, the partial splenic embolization approach has been widely used and approved worldwide, resulting in good outcomes and reduction in the number of reports of major complications [36, 47, 49]. Partial splenic embolization is a widely accepted technique for treating hypersplenism in children [35, 37, 42, 43, 50, 51] and in many health care centers partial splenic embolization has emerged as the treatment of choice for children with hypersplenism [37]. In partial splenic embolization, the arterial blood supply at the level of the end arterioles of the spleen is reduced through injection of embolic material, and the resulting ischemic necrosis of splenic parenchyma results in a decrease in size of the spleen and reduction of the hypersplenism, a decrease of portal venous inflow and reduction of gastroesophageal varices [35, 37, 52] (Fig. 4).

The unique anatomical arterial characteristics of the spleen make it an ideal organ for partial embolization [49, 53]. Small splenic segments can be identified based on the terminal arterial blood supply [49, 54]. Therefore, embolization of a certain artery does not affect the remaining splenic parenchyma. The left gastroepiploic artery and other gastric branches originate from the terminal branches of the splenic artery, and branches for the pancreas originate from the middle segment of the splenic artery [49, 54]. In pre-embolization angiography, it is crucial to visualize these branches for the pancreas [54, 55]. There can, of course, be variation in origin, course and terminal branching of the splenic artery, and the interventional radiologist must take these variations into account [35, 49, 54].

In the pediatric population, partial splenic embolization is performed under general anesthesia or deep sedation by an interventional radiologist. Usually, the percutaneous approach is through the femoral artery using the Seldinger technique under sterile conditions.

Embolization is carried out according to guidelines based on Spigos et al.’s [48] recommendations (i.e. antibiotic prophylaxis, pain control, limited volume embolization). Prior to partial splenic embolization, digital subtraction angiography of the celiac trunk and the splenic artery is obtained to determine the precise anatomy of the splenic arterial branches and to identify the target arteries and visualize branches to other tissues [35, 42, 51, 52, 54–58].

Partial splenic embolization can be performed using two methods: selective catheterization and embolization, or non-selective embolization [49, 54]. In selective partial embolization, only a few targeted distal branches of the splenic artery are completely embolized. The extent of embolization and the estimation of remaining viable splenic volume can be assessed on parenchymal phase angiograms [49, 54]. Using the non-selective method, the embolic materials are injected more proximally in the main splenic artery, but beyond the origin of the pancreatic branches. Embolization is performed until parenchymal blush is reduced [38, 49, 54].

In partial splenic embolization the most used embolic agents are gelatin sponge pledgets, polyvinyl alcohol particles and trisacryl gelatin microspheres. These agents can be injected in a suspension containing a contrast agent and antibiotics [36, 54].

According to the literature, in cirrhotic patients the ideal splenic volume target for partial splenic embolization should be 50–70%; a higher incidence of complications is described when embolization involves more than 70% of the total splenic volume [35–37, 40, 42, 51, 54, 58].

Data show that partial splenic embolization has, over time, become a safe procedure for pediatric patients if certain criteria are met. These criteria include: procedure performed by an experienced interventional radiologist, maximum of 70% spleen infarction, respect of aseptic conditions and use of antibiotics and highly effective analgesia to prevent pulmonary complications [35, 38, 40, 44, 47, 50, 58]. The most common major complications are pleural effusion, ascites, portal vein thrombosis and splenic abscess [36, 37, 54].

Following partial splenic embolization, a so-called post-embolization syndrome is observed in most patients, at a frequency of 73.4%; this condition is considered a minor complication and consists mainly of fever, nausea, left upper quadrant pain and perisplenic fluid collection, and these symptoms are usually controlled with antibiotic prophylaxis, narcotics and antiemetics [35–37, 54]. Partial splenic embolization preserves a residual functional spleen as a protection against infections [37].

In summary, recent data indicate that partial splenic embolization is a safe and effective alternative procedure to splenectomy in the pediatric population with splenomegaly and hypersplenism secondary to portal hypertension. Partial splenic embolization can be used to improve liver function and hematological status, to prevent variceal hemorrhage and to treat hepatic encephalopathy [35, 37, 44, 46, 47, 50, 53, 59].

## Transjugular intrahepatic portosystemic shunt

In adults with cirrhosis, complications of portal hypertension have traditionally been managed with endoscopic variceal ligation [35]. However, transjugular intrahepatic portosystemic shunt (TIPS) placement also represents a common procedure for treating the complications of portal hypertension, especially to avoid variceal bleeding while awaiting liver transplantation [60, 61]. Despite the different etiologies of liver disease in adults and children, manifestations of portal hypertension are similar and include encephalopathy, variceal bleeding and ascites [62]. Indications for TIPS in both adults and children include uncontrolled variceal hemorrhage, refractory ascites, hepatic pleural effusion, hepatorenal syndrome, veno-occlusive disease and Budd–Chiari syndrome [63, 64].

Transjugular intrahepatic portosystemic shunt placement is considered difficult in children. The technique can be challenging in situations of distorted hepatic vascularization, modified liver anatomy or segmental liver grafts, where advanced skills are required. Children with < 10 kg of body weight might not tolerate TIPS because of the size of the device (manufactured for adults) and the hemodynamic changes that follow the placement of a large shunt, which can cause a remarkable increase in the systemic venous return to the right heart. Perhaps for these reasons, few children have been proposed and undergone this procedure [65–72].

However, the procedure is not impossible in children, as reported by Izaaryene et al. [72], who described a successful TIPS placement in a 3-month-old weighing less than 10 kg and with rapidly progressing refractory ascites secondary to portal fibrosis of unknown origin. Pediatric TIPS placement generally parallels the technique used in adults, incorporating occasional modifications dictated by patient size and anatomy [62]. Although the Rösch-Uchida Transjugular Liver Access Set (Cook Medical) is the most used set, access sets modified for pediatric patients are available and use an 18-G Colapinto needle and 7-Fr sheath (Cook Medical); however, they are not amenable for delivery of expanded polytetrafluoroethylene (ePTFE)-covered stents. Intravascular US is another tool available for TIPS creation in both children and adults, and steerable side-firing intravascular US probes are available in 8-Fr and 10-Fr sizes, which are small enough for venous access in children [62].

**Fig. 5:**
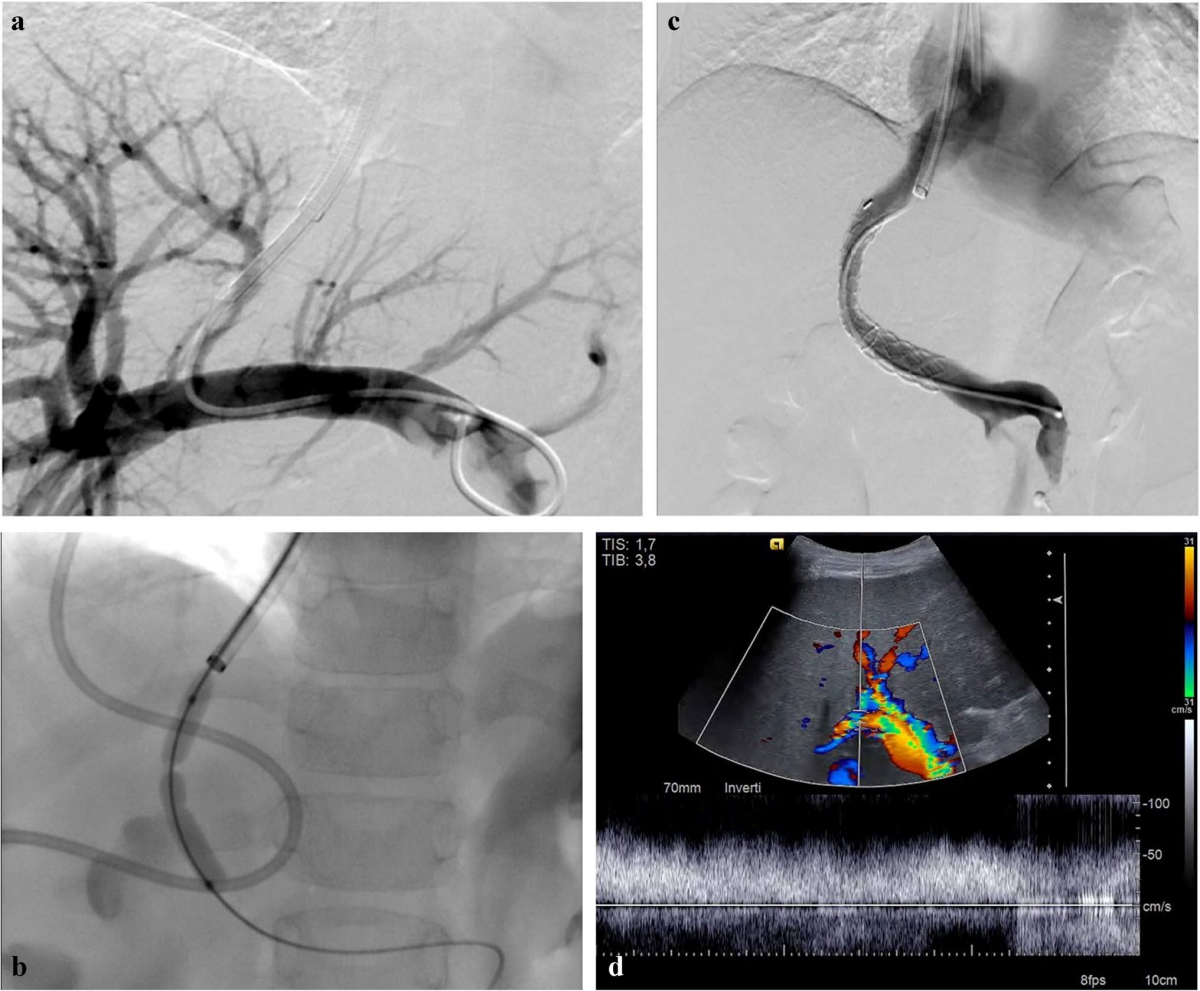
Transjugular intrahepatic portosystemic shunt (TIPS) placement in a 9-year-old girl with liver cirrhosis. **a** Posteroanterior (PA) portal venogram. **b** PA fluoroscopy image shows balloon dilation of the intrahepatic tract. Note the proximal and distal balloon notches indicating the length of the hepatic tract. **c** Portal PA venogram after stent positioning shows good flow through the TIPS. **d** Three-month follow-up axial Doppler US shows good patency of the TIPS with no late complications

All procedures are performed under general anesthesia. A 9-Fr or 10-Fr sheath (Terumo Europe) is inserted into the right internal jugular vein. A 5-Fr Cobra catheter (Terumo Europe) is then wedged centrally in the liver in the right or middle hepatic vein and a wedged hepatic vein phlebography is performed to identify the portal vein. A 16-G Colapinto needle (Cook Medical) is used to puncture the portal vein under continuous US monitoring. After entering the portal vein, a 0.035-in. wire is advanced followed by a 5-Fr catheter (Terumo Europe) into the portal vein. A portal venogram (Fig. 5) is obtained and, at the same time, pressure measurements are obtained from the portal vein and right atrium. Balloon dilation and stent positioning (Fig. 5) are then carried out, with the stent for bridging the shunt varying according to the operator’s choice. Stent diameters are selected by the operator in relation to the child’s height and weight and size of the portal vein. The goal of stent placement is to have a gentle curve with the distal end extending 2 cm into the portal vein and the proximal end extending near to the hepatic vein/inferior vena cava confluence [73].

Ghannam et al. [73] reported a successful TIPS placement in 20/21 children. Eighty percent of these children had a reduction of the portosystemic gradient to ≤ 12 mmHg, with no recurrence of variceal hemorrhage or refractory ascites, corresponding to a clinical success rate of 100% [73]. The technical success of this study mirrors reported rates in the literature except for one report from China [65, 66, 68]. The single failed TIPS in this series was secondary to complete intrahepatic and extrahepatic portal vein occlusion.

Minor and major complications are common after TIPS placement and include extracapsular puncture, shunt occlusion and dysfunction, recurrence of ascites or variceal hemorrhage, intraperitoneal hemorrhage and hepatic encephalopathy. In Ghannam et al.’s [73] report, no intra- or immediate post-procedural complications were described according to the Society of Interventional Radiology guidelines [74, 75], mirroring reported outcomes in the two prior larger series [65, 68]. The 30-day complication rate in the Ghannam report is similar or slightly lower than that of the prior reports in the literature [65–68, 76, 77].

Hepatic encephalopathy and stent malfunction are two of the most common post-procedural complications of TIPS placement [78]; as compared to endoscopic therapy, hepatic encephalopathy rates are higher after TIPS placement and in this series, hepatic encephalopathy occurred in 48% of children who underwent TIPS procedure. In Bertino et al. [62], TIPS creation was successful in 57/61 (93.4%) attempts; hemodynamic success rate was 94% (47/50) and overall clinical success rate was 80.7% (46/57).

The major complications rate reported by Bertino et al. [62] was 8.2% (with hemoperitoneum requiring resuscitation in 2/5 and death in 3/5); the minor complications rate was 21.3%. Di Giorgio et al. [65] reported that no patient developed major complications following the procedure, confirming that TIPS is safe in children.

To summarize, TIPS creation in children and adolescents is a technically feasible and efficacious procedure with a low complication rate for the treatment and prevention of uncontrolled variceal hemorrhage and refractory ascites and should not only be considered as a bridge to transplantation, but also as an effective and less invasive alternative to surgical vascular shunts.
